# Dr. Benjamin M. Griffith

**Published:** 1898-11

**Authors:** 


### OBITUARY.

Dr. Benjamin M. Griffith, of Springville, Ill., died at his residence in that city, September 24, 1898, aged 68 years. Doctor Griffith was born in Shelbyville, Shelby county, Kentucky, April 14, 1831, and moved with his father when young to Auburn, Lincoln county, Mo. He began the preparatory study for medicine at a school in Louisiana, Mo., in 1853, and was graduated in 1859 from the St. Louis Medical College.

In 1865 he located at Springfield, where he continued to reside and practise his profession until his last illness.

Doctor Griffith was a member of the city board of health for a number of years and always took an active interest in the city's sanitary condition. He was a member of the Illinois State Medical Society, the American Medical Association and the National Confederation Medical Examining and Licensing Boards, having served one year as secretary of the latter body.

In 1892 Dr. Griffith was appointed consulting physician of St. John's Hospital, in which position he served for years with distinction. He was at the head of the hospital staff when he died.

Dr. Griffith was a man of strong character, a conscientious and able physician and an accomplished sanitarian. He is survived by a widow and two children—Dr. B. B. Griffith, of Springfield, and Mrs. S. J. Pitner, of Jacksonville.

